# Approaches and Challenges in the Management of Multiple Myeloma in the Very Old: Future Treatment Prospects

**DOI:** 10.3389/fmed.2021.612696

**Published:** 2021-02-25

**Authors:** Natasha Mupeta Kaweme, Geoffrey Joseph Changwe, Fuling Zhou

**Affiliations:** ^1^Department of Hematology, Zhongnan Hospital, Wuhan University, Wuhan, China; ^2^Department of Cardiac Surgery, Qilu Hospital, Shandong University, Jinan, China

**Keywords:** multiple myeloma, older patients, frailty, geriatric assessment, future therapies

## Abstract

The increasing incidence of geriatric patients with multiple myeloma has elevated concerns in clinical practice. While the introduction of novel therapeutic agents has substantially improved outcomes in younger patients with myeloma, poorer outcomes remain in older patients. Managing older patients requires a multidisciplinary team approach to consider factors that may influence both treatment selection and outcomes. Aging is associated with remodeling of vital organs, physiological downregulations of basal metabolism, susceptibility to multiple comorbidities with ultimate frailty, thereby contributing to the underrepresentation and exclusion of very old patients from clinical trials. Therefore, timely confirmation of a precise diagnosis is crucial for prompt initiation of treatment if the desired outcome is to be achieved. Adequate and judicious assessment using comprehensive geriatric assessment tools minimizes toxicities and treatment discontinuation. Initiating treatment with combinational therapy requires knowledge of indications and anticipated outcomes, as well as individualized therapy with appropriate dose-adjustment. Individualized therapy based on good clinical acumen and best practices obverts unwanted polypharmacy, preventing iatrogenic harm. This review will therefore address the approaches and challenges faced in managing myeloma in geriatric patients aged 80 years and older, highlighting recommended therapeutic strategies and future prospective regimens.

## Introduction

Multiple myeloma (MM) is an incurable plasma cell neoplasm and is largely a disease of older adults. The median age at diagnosis is 70 years (1). At initial diagnosis, approximately two-thirds of patients are older than 65 and only one-third are over 75 years of age (2). Because of an aging population, the prevalence of MM is projected to rise substantially at an estimated 80% per year in the next 20 years (3). According to the recent development of novel therapeutic agents, enhancement in the safety of Autologous-hematopoietic Stem Cell Transplantation (ASCT) and the availability of hospice care have significantly improved survival in younger patients (65 years). However, these novel approaches have shown an insignificant improvement in older patients (75 years) and shown poorer outcomes in very old patients (80 years) (4–8). Consequently, a continued understanding of the factors and challenges leading to such poor outcomes in the very old patient category is needed.

Aging is gradual and progressive which eventually leads to reduced physical and physiological functions of the body and vital organs. This decline in physiological function and organ dysfunction results in increased susceptibility to comorbidities in this population (9). The presence of comorbidities precludes older adults from meeting the eligibility criteria for inclusion and representation in randomized clinical trials, thus resulting in the difficulty to assess treatment outcomes (10, 11). Older adults have a higher risk of frailty, therefore, more vulnerable to Adverse Events (AEs) in the presence of minor stressors. Furthermore, cumulative deficits in multiple physiological systems result in reduced drug tolerance, dose reductions, and reduced quality of life (12).

At the initial diagnosis of MM, close to one-third of older patients are frail (3, 13). The heterogeneous nature of older patients (1, 3, 14) ranging from being fit to frail, requires that each patient be individually assessed before initiation of treatment. Making an adequate assessment is essential to avoid undertreating very fit patients and over-treating frail patients. Thus, Comprehensive Geriatric Assessment (CGA) tools have been created and designed to help clinicians in both diagnosis and treatment of older patients. Although a specific CGA tool created for categorizing myeloma patients into various fitness groups is available (14), there is limited evidence on CGA outcomes, specifically among patients with hematological malignancies (7).

The management of older patients with MM requires a consideration of all influencing factors including environmental and social factors. Hence, limited access to healthcare, healthcare providers and social care have been shown to impact patient outcomes. This review will therefore address the approaches and the main challenges faced in managing geriatric patients aged 80 years and older, highlighting recommended therapeutic strategies and future prospective regimens.

## Delayed Diagnosis and Diagnostic Uncertainty

Delayed diagnosis of MM in older patients is attributed to several factors including inadequate understanding of basic pathophysiology and clinical manifestations, both symptomatic and asymptomatic. The presence of myeloma-related organ dysfunction often accelerates diagnosis (15). In most patients, myeloma is preceded by an asymptomatic Monoclonal Gammopathy of Undetermined Significance (MGUS), which is a premalignant plasma cell disorder. Kyle et al. reported that in patients who are 80 years or older diagnosed with MGUS, the lesion remained benign, however, 4–5% exhibited a paraprotein (16). Furthermore, the incidence of MM in this age group was reported to be 40 per 100,000 (5). The presence of MGUS lesions with unrelated organ dysfunction should elicit a thorough follow-up for possible MM.

Smoldering MM (SMM) is an intermediate stage with a propensity to progress into symptomatic MM due to the increased plasma cell burden. Both MGUS and SMM are ordinarily asymptomatic and distinguished from each other by the measure of secreted monoclonal proteins and/or infiltration of clonal plasma cells in the bone marrow (17).

### Miscellaneous Manifestation of MM

Patients with MM may present with clinical signs and symptoms, including unexplainable anemia and fatigue, bone pain or pathological fractures, spinal cord compression, hypercalcemia, renal insufficiency, recurrent infections, and rarely hyperviscosity symptoms (15).

Hypercalcemia results from an increase in either osteoclastic bone resorption or renal tubular calcium reabsorption. Although not common, hypercalcemia was present in 13% of subjects at diagnosis. Renal impairment is prevalent, occuring in 50% of patients at diagnosis (18), and is attributed to infiltration of the renal parenchyma by various plasma circulating cellular products, concurrent amyloidosis, hypercalcemia and recurrent urinary tract infections. Anemia (73%) is attributed to a reduction in erythropoietin production and suppressive effects of excess cytokines on erythropoiesis (19). Bone involvement (53%) manifests as either pain or pathological fractures due to bone infiltration by plasmacytoma, osteolytic bone lesions and osteoporosis (20).

In younger patients with symptoms and a paraprotein or light chain excess, the diagnosis of MM often quickly progresses. On the contrary, older patients may have symptoms attributed to other etiologies, requiring a detailed evaluation before confirming a diagnosis. Hypercalcemia may be attributed to parathyroid hormone or not. Non-parathyroid hormone-related causes include thiazides and lithium, malignancies involving bone metastases, endocrine disorders, and granulomatous diseases. Given the high prevalence of comorbidities in the very old, 60% of patients have age-related Chronic Kidney Disease (CKD), Hypertension (HTN), diabetes, and cardiovascular disease which precipitate renal injury with the use of NSAIDs and diuretics, dehydration, and infections can precipitate acute renal failure in older adults.

Anemia affects ~25% of older patients and can be attributed to chronic disease, iron deficiency, vitamin B12, folate deficiency, CKD, and other diseases. Bone pain may be malignant or non-malignant related (i.e., osteomalacia and osteomyelitis). Therefore, the diagnosis and confirmation of MM disease in this age group should be made only after excluding alternative diagnoses (1, 2). Koshiaris et al. reported a delay of ~6 months from the time of symptoms presenting to the diagnosis of MM, with over 50% of patients requiring three visits to a general practitioner before a referral to a first-level hospital is made (21). This significant delay in assessment and early diagnosis leads to delayed treatment and disease progression.

The Revised International Myeloma Working Group criteria (R-IMWG) for the diagnosis of symptomatic MM are defined by the presence of clonal bone marrow plasma cells ≥10% or biopsy-proven extramedullary plasmacytoma with any one or more of the listed myeloma defining events; (1) evidence of end-organ damage attributed to an underlying plasma cell proliferative disorder, (2) one or more biomarker of malignancy (Table 1) (17, 22). The revised criteria comprise three biomarkers and each is associated with an ~80% risk of progression from smoldering to symptomatic organ damage. In SMM, the presence of serum monoclonal protein (IgG or IgA) ≥3 g/dL or urinary monoclonal protein ≥500 mg per 24 h and or clonal bone marrow plasma cells 10–60%, and the absence of myeloma-defining events or amyloidosis is established as the diagnostic criteria (23). Both criteria must be met for the diagnosis of SMM.

**Table 1:** Revised IMWG diagnostic criteria for symptomatic MM.

1. End-organ damage attributed to plasma cell infiltration CRAB (HyperCalcemia, Renal impairment, Anemia, and Bone lesions) criteria	
Hypercalcemia Renal failure Anemia Bone disease	Serum calcium >0.25 mmol/L (>1 mg/dL) above normal range or >2.75 mmol/L (>11 mg/dL). Serum creatinine > 1.77 μmol/L (> 2 mg/dL) or creatinine clearance <40 mL/min Hemoglobin <100 g/L, (12.5 mmol/L) or >20 g/L (1.25 mmol/L) below the lower limit of normal. Lytic bone lesions, severe osteopenia, or pathological fractures; one or more notable osteolytic bone lesions on plain XR/CT/PET-CT scan.
2. Biomarkers of Malignancy ≥60% Clonal bone marrow plasma cells involved: uninvolved sFLCr ≥100 > 1 focal lesion ≥5 mm on MRI	

*XR, x-ray; CT, computed tomography; PET-CT, positron emission tomography-computed tomography; sFLCr, serum free light chain ratio; MRI, magnetic resonance imaging*.

Generally, SMM progresses to MM at a rate of 10% per year following the first 5 years of diagnosis, 3% over the following 5 years, and 1.5% per year henceforth. The cytogenetic abnormalities determined at the time of diagnosis and cytogenetic changes during the disease course influence the rate of progression from MGUS or SMM to MM (15). Accordingly, MM diagnostic criteria have been revised to identify patients at high risk of rapid progression from smoldering to symptomatic myeloma. However, in the very old, the risk stratification for progressive disease based on the established criteria might not be relevant due to their advanced age.

### Standard Investigative Work-Up for MM

The recommended investigations for the diagnosis of MM in the very old include; (i) a detailed medical history and physical examination, (ii) routine blood tests (complete blood count, biochemistry analysis, serum and urinary protein electrophoresis with immunofixation, monoclonal protein quantification, and serum-free light chain ratio), (iii) bone marrow evaluation (trephine biopsy and aspiration for cytogenetic analysis or FISH panel) (1), and (iv) radiological imaging with X-ray, MRI, PET-CT to identify myeloma-related bone lesions, assess spinal cord compression and exclude multifocal plasmacytomas. Further, serum albumin, β2-microglobulin, and lactate dehydrogenase are recommended to assess tumor burden and disease prognosis. The diagnostic criteria and investigations required for the diagnosis of symptomatic MM in older and very old patients are the same for younger patients. However, clinicians should distinctly determine that the disease presentation fulfills the criteria for anti-myeloma therapy.

Bone marrow sampling is critical in confirming a diagnosis of myeloma. Although it is tolerated in older adults, caution must therefore be given to frail patients with a relative risk of bleeding and underlying osteoporosis. This may lead to challenges in performing a bone marrow biopsy. Diagnosis of high-risk cytogenetic abnormalities such as chromosome 17p (TP53 deletion), t (4;14), t (14;16), non-hyperdiploid, and gain(1q) assessed by fluorescence *in situ* hybridization (FISH), are associated with poor outcomes and prognosis in older patients and regardless of age. The IMWG consensus panel on FISH recommends testing for the presence of del(17p), t (4;14), and possibly t (14;16). t (11;14) and t (6;14) are classified as standard-risk (24). Application of risk factors, such as cytogenetic abnormalities, enhances therapy precision across all myeloma patient groups. However, in patients ≥80 years, patient outcomes cannot be explained by a higher prevalence of adverse cytogenetic profiles, which is less apparent in very old adults due to a lower incidence of t (4;14) (25).

## Geriatric Assessment and Vulnerability Scores

Since older adults are a heterogeneous population, their aging and frailty status should be considered when placed in clinical trials. It has been demonstrated that a strong relationship between age and the number of comorbidities exists, i.e., one-third of patients between the age of 65 and 79 years have at least one comorbidity. In patients ≥80 years, the percentage risk of the number of comorbidities was increased to 70% (26). Studies have shown improved Progression-Free Survival (PFS) in patients ≤75 years, but not in the very old ≥80 years. This makes it vital to undertake a geriatric assessment before the commencement of treatment.

While CGA is standard practice for geriatric patients, a full CGA is time-consuming and challenging to use in everyday practice (27). However, a specific CGA has been developed mainly for older myeloma patients, which categorizes them according to frailty status with the view to predicting outcomes (14). The CGA tools are Katz and Akpom's basic activities of daily living (ADL), Lawton and Brody's Instrumental Activities of Daily Living (IADL) scale, and the Charlson Comorbidity Index (CCI). ADL and IADL scores assess activities of self-care and independent living skills, whereas CCI assesses the number and severity of comorbidities. Table 2 illustrates practical uses of GA tools in evaluating comorbidities, cognitive and functional status in older patients with hematological malignancies.

**Table 2:** Practical use of ADL, IADL, and CCI tools in older patients with hematological malignancies.

**Selected CGA tools**	**Function**	**Use in clinical valuation of older patients**	**References**
ADL IADL	Cognitive, functional assessment	Predict OS and AEs in older NDMM patients Enhance functional assessment in older patients with AML Determine frailty before treatment in older patients with hematological malignancies Determine treatment goals in older hematological patients, i.e., CLL	Zhong et al. (28) Carbonell and Chauffaille (29) Abel and Klepin (30) Shanafelt (31)
CCI	Comorbidity assessment	Predict OS in older NDMM patients. Determine the effect of comorbidity indices in older MM patients. Evaluate the impact of comorbidity on survival in NDMM patients Predict chemotherapy use and OS in older lymphoma patients Evaluate the impact of comorbidity on survival in Post-ASCT MM patients Evaluate the influence of comorbidity on OS and EFS in MDS patients	Kim et al. (32) Bila et al. (33) Gregerson et al. (34) Shah et al. (35) Veljanovska et al. (36) Sperr et al. (37)

*CGA, Comprehensive Geriatric Assessment; ADL, Activities of Daily Living; IADL, Instrumental Activities of Daily Living; CCI, Charlson Comorbidity Index; OS, Overall Survival; AEs, adverse events; NDMM, Newly Diagnosed Multiple Myeloma; CLL, Chronic Lymphocytic Leukemia; ASCT, Autologous Stem Cell Transplant; EFS, Event-Free Survival; AML, Acute Myeloid Leukemia*.

ADL, IADL, and CCI were combined with age to categorize older patients into three groups; fit, intermediate, and frail. Although the cutoff age for frailty is 80 years (14), patients can be categorized as frail when there is a functional decline on either ADL, IADL, or presence of comorbidities regardless of age. The effective use of the scoring systems was demonstrated in three multicenter trials where different methods of assessment were performed before treatment of (*N* = 869 MM patients, with a median age of 74 years, of which 46% were ≥5 years) (38, 39). Performance status on GA predicted survival, non-hematologic AEs and treatment discontinuation in older patients. Consequently, a GA is and should be mandatory as drug efficacy and effects of toxicity are significantly diverse in fit, unfit and frail patients.

Several GA tools are valuable in many cancers, but most of the tools are not specific to myeloma. Notwithstanding, the IMWG devised a scoring system that categorizes patients according to fitness based on the total additive score and more importantly, predicts mortality risk. The tool was specially designed for clinical evaluation, cross-comparison of clinical trials, and frailty measurement in designing future trials. The scoring system is based on age, ADL, IADL and CCI, which results in a score ranging from 0 to 5 (40). In a retrospective study, data from the Czech Myeloma Group Registry of Monoclonal Gammopathies was used to validate the IMWG frailty score and Revised International Staging System (R-ISS) indices for risk stratification in patients with MM in clinical practice. The results concluded that the prognostic value of the IMWG and R-ISS risk stratification indices apply to patients with MM in real-world settings (41).

Other GA tools include the Karnofsky Performance Status (KPS) or the Eastern Cooperative Oncology Group (ECOG), whose performance scores mainly focus on the degree of functionality and correlate with overall survival (OS) in myeloma patients (42). However, more definitive predictive scores are still needed to guide clinical decisions because performance scores alone, do not accurately predict outcomes in the very old (43). Another alternative CGA tool useful for assessing comorbidities is the Freiburg Comorbidity Index (FCI). FCI is specifically validated for MM and includes patients' KPS, renal and pulmonary function (FEV1/FVC) (44). In a cohort study of 466 MM patients to validate FCI in combination with ISS, patients were divided into three distinct groups: low-risk (FCI 0 and ISS I-II), intermediate-risk (all remaining), and high-risk (FCI 1-3 and ISS III) with OS probabilities at 5-years of 85, 74, and 42%, respectively (*P* < 0.0001). FCI is a reliable comorbidity index to consider in future trials.

The impact of IMWG scores on clinical outcomes was validated using data from the univariate and multivariate analyses of an external cohort of 125 newly diagnosed MM patients. The analyses demonstrated that cytogenetics, impaired renal function, lung function, and KPS improved the prediction of fit, intermediate-fit, and frail patients leading to the development of the “revised” myeloma comorbidity index (R-MCI). R-MCI incorporates relevant risk factors and MM-related cytogenetics (40). The R-MCI was evaluated in a cohort of 801 patients newly diagnosed with MM, with 13% ≥75 years. The study proved the benefit of R-MCI on the accurate assessment of patients' physical conditions and simple clinical applicability (45).

The significance of a complete GA in older and very old patients cannot be overemphasized. However, research to develop and validate scoring systems that will guide and rationalize treatment decisions in this age group are still required. The IMWG incorporates older patients based on age groups ≤75 years, 76–80 years, or >80 years and identifies patients who might have poor outcomes and increased incidence of grade 3/4 AEs (46).

## Treatment Choice and Dose Intensity

This review has established that treating MM in the very old requires understanding the indications and possible outcomes of treatment. The decision to initiate combination therapy and aggressiveness requires a multidisciplinary team approach, considering all available diagnostic and clinical information, a complete CGA, and in consultation with the patient and or their family.

MM is often preceded by precursor states of MGUS and SMM (47, 48). These represent a continuation of tumor burden without symptoms or end-organ damage. Patients with MGUS are usually monitored in primary healthcare centers with pre-agreed guidelines for re-referral if the need arises (49). SMM is stratified based on Mayo Clinic criteria into low-risk, intermediate-risk, and high-risk. For patients with high-risk SMM, clinical trials are highly recommended or close observation for those not enrolled in trials. On the other hand, for clinically stable low-risk SMM patients, less frequent monitoring is required. Treatment is recommended for patients with active myeloma.

Novel agents in MM treatment have not significantly improved outcomes in very old patients likely because of increased comorbidities, frailty, and vulnerability to AEs associated with high dose chemotherapy. As a result, very old patients are rare candidates for high dose therapy and ASCT. The decision for ASCT requires a critical assessment of the overall health status of the patient, performance status, cardiac, pulmonary, and renal function, myeloma risk features, disability and frailty, and psychosocial/economic evaluation (9). Previously, the age cutoff adopted to determine the eligibility of ASCT was 65 years which eventually was extended to 70–75 years in clinical practice. The European Society of Medical Oncology (ESMO) recommended ASCT up to the age of 70 years. According to ESMO, patients >80 years do not meet the eligibility criteria for ASCT. Conversely, the National Comprehensive Cancer Network (NCCN) did not set an age cutoff (25).

Studies have revealed that patients >65 years have a higher risk of post-transplantation complications and prolonged hospitalization compared to younger patients (25, 50). Despite this, subsequent clinical trials demonstrated that prolonged hospitalization and post-transplant complications did not translate into higher treatment-related mortality compared to younger patients, and this was attributed to the reduction of the conditioning regimen. For the foregoing reason, the data presented supports ASCT in older patients but with careful patient selection, fitness assessment, availability of social support to minimize complications and treatment-related mortality (51).

The inclusion of older adults in clinical trials could provide a platform to assess post-transplantation complications and outcomes and establish optimal transplantation regimens. For over a decade, Melphalan-Prednisone (MP) was the mainstay treatment for transplant-ineligible, older MM patients. The introduction of IMIDs, PIs, and monoclonal antibodies (mAbs) has proved safe and effective.

### Proteasome Inhibitors

Bortezomib-based regimens are the first class of PIs approved as front-line therapy in treating older MM patients. Previously, studies such as the phase III VISTA trial and phase IIIB UPFRONT trial investigated the multi-drug combinations of bortezomib (Velcade) with other anti-myeloma agents for transplant-ineligible older patients. The phase III VISTA study included 682 patients aged ≥81 years (median age 71) with 30% of patients aged ≥75 years. The study investigated the outcome benefit of bortezomib plus MP compared to MP exclusively as front-line treatment in older patients unfit for ASCT. VMP demonstrated significant OS benefit compared to MP exclusively. The addition of bortezomib to MP did not significantly affect the safety and efficacy profiles. The rates of AEs were higher in the VMP arm, with peripheral neuropathy (PN) reported more frequently. Generally, VMP was well-tolerated with manageable toxicities (52).

Velcade use in older patients ineligible for transplant was demonstrated in the UPFRONT trial. The study compared three bortezomib-based regimens, VD and VTD, with VMP. This was the first study on multiple bortezomib-based regimens to reflect patient diversity in the ‘real-world’ clinical setting. Five hundred and two patients were included, with a median age of 73 years. Of the sample, 42% of the patients were ≥75 years, and 18% ≥80 years. The trial revealed no significant difference among the three regimens for either end-point of the spectrum. After 42.9 months' median follow-up, median PFS with VD, VTD and VMP was 14.7, 15.4, and 17.3 months, OS 49.8, 51.5, and 53.1 months; global P = 0.46 and P = 0.79 and ORR 73, 80, and 70%. All three regimens showed substantial benefits and good outcomes. The most common AE was PN with a safety profile consistent with known toxicities for the component drugs (10).

Recently, bortezomib with lenalidomide (R) and dexamethasone was investigated for transplant-ineligible newly diagnosed MM patients. The randomized phase III SWOG S07777 trial compared the efficacy of VRd to Rd alone in 529 newly diagnosed myeloma patients. The study showed significantly superior response rates, PFS and OS, and a favorable risk-benefit profile with VRd compared to the approved front-line regimen of Rd. In the subgroup analysis, VRd improved OS in patients older than 75 years with a median OS of 63 vs. 31 months with Rd alone (53).

The improved benefit of bortezomib plus thalidomide (T) plus dexamethasone (VTd) and bortezomib plus cyclophosphamide (C) plus dexamethasone (VCd) have shown superior response rates in transplant-ineligible patients and are therefore valuable alternatives to VRd (15). Chan et al. reported on the benefits of VCd as a front-line treatment for newly diagnosed older patients (≥70 years) ineligible for transplant. Data from the retrospective multicenter analysis showed superior response rates and improved outcomes with VCd in older patients with a pre-planned switch to VTd prolonging event-free survival (EFS) (54). The toxicity profile was difficult to evaluate due to the retrospective nature of the study. However, treatment discontinuation due to toxicity was similar to the phase IIIB UPFRONT study.

The comparative efficacy of VMP with or without daratumumab vs. VMP exclusively in NDMM was demonstrated by a propensity score matching analysis of the ALCYONE and VISTA phase III studies. Results demonstrated that a lower intensity VMP regimen such as in ALCYONE had a favorable benefit/risk profile compared with the VISTA VMP regimen, and D-VMP significantly improved efficacy vs. VISTA VMP (55). Grade 3/4 PN was significantly lower in both arms of the ALCYONE study vs. VISTA VMP. ALCYONE D-VMP had significantly improved efficacy compared to VISTA VMP, supporting modified bortezomib in VMP-D for transplant-ineligible NDMM.

Previously, bortezomib was associated with PN that could cause severe pain to patients. To minimize the incidence of neuropathy, the regimen was modified to once-weekly SC bortezomib instead of twice-weekly intravenous (56), making regimens like VRd, VCd, and VTd more tolerable. The adjusted regimen is recommended in very old patients to minimize drug discontinuation and further dose reductions (56, 57). In newly diagnosed older patients with pre-existing comorbidities, initial therapy with VRd administered for ~8–12 cycles is recommended, followed by lenalidomide maintenance (23).

### Immunomodulatory Drugs

#### Thalidomide-Based Regimens

A meta-analysis of six clinical trials demonstrated the survival benefit of thalidomide plus MP compared to MP alone in untreated older patients (58–63). One of the randomized phase III trials specifically investigated the use of MPT in patients older than 75 years with NDMM (63). A pulled meta-analysis of patient data collected from the six clinical trials by Fayers et al. reported that the additive effect of thalidomide to MP on OS varied across all the trials. Results showed a significant benefit in OS with an increased median OS of 6.6 months, from 32.7 months (MP) to 39.3 months (MPT) improved response rates and depths of response (ORR 59% for MPT vs. 37% for MP; *P* < 0.001) and prolonged PFS of about 6 months in most of the trials (64, 65). In the IFM 01/01 (phase III) trial, which specifically investigated the use of MPT in NDMM patients older than 75 years, results showed a prolonged PFS of 18.5 to 24 months (*p* = 0.001) and OS of 29.1 to 44.0 months (*p* = 0.028) also favoring the MPT regimen (63).

Although the MPT regimen improved response rate, OS, and PFS and is effective as a first-line treatment of MM in older patients, it is poorly tolerated at high doses due to increased risk of toxicity. Previously, due to drug side effects such as Venous Thromboembolism (VTE), PN, constipation, cardiac events, fatigue and drowsiness, thalidomide was prematurely stopped or the dosage was reduced. Thus, it is strongly advised to assess tolerability and administer an appropriate dosage in very old patients (i.e., 50–100 mg once daily max.). Additionally, thalidomide should be avoided or used cautiously in older patients with renal failure. Trials are ongoing to prove that thalidomide may be an effective, well-tolerated alternative front-line therapy (66).

Lenalidomide, a second-generation IMiD, has shown higher potency and lesser toxicity compared to thalidomide. In the MM-015 study, the benefit of melphalan, prednisone, and lenalidomide (MPR) with lenalidomide maintenance (MPR-R) or without vs. standard MP treatment in patients ≥65 years with NDMM, ineligible for transplant was investigated. Results showed a significant improvement in PFS by the addition of lenalidomide during induction and maintenance phase: MPR-R (31 months), MPR (14 months; HR, 0.49; *P* < 0.001) and MP (13 months; HR, 0.40; *P* < 0.001). However, this benefit was disregarded in older patients because of hematological toxicities. The most common hematologic grade 4 AEs during induction was neutropenia which occurred more in the lenalidomide groups, with little evidence of cumulative toxic effects associated with lenalidomide maintenance (67, 68).

Lenalidomide with dexamethasone is a recommended standard regimen for the initial treatment of older MM. To investigate the benefit of low-dose dexamethasone or high-dose dexamethasone with lenalidomide, The ECOG conducted a study of 445 patients randomly assigned to either the low-dose dexamethasone group [40 mg once weekly (Rd)] or high-dose dexamethasone group [40 mg for 4 days on, 4 days off (RD)] with a primary end-point of response rate after 4 cycles (69). The trial showed better response rates for patients who received RD, but at high toxicity. As a result, the trial was stopped, and all patients switched to low-dose therapy. Because low-dose therapy yielded a better OS at 1-year with lower toxicity, it is preferred and recommended for NDMM. Currently, Rd is recommended mainly for patients unable to tolerate triplet drug regimens due to advanced age, comorbidities, and poor performance status.

For transplant-ineligible MM patients, trials are ongoing to establish safer and effective therapies while maintaining minimal toxicity. The Frontline Investigation of Lenalidomide plus Dexamethasone vs. Standard Thalidomide (FIRST) is one of the most extensive and reliable trials to investigate the benefit of MPT compared with Rd 18 cycles and Rd continuously in transplant-ineligible patients with NDMM. The trial included 1,623 patients, aged ≥18 years, randomized into one of the three treatments arms: Rd continuous, Rd18 (Rd for 18 cycles [72 weeks]), or MPT (for 12 cycles [72 weeks]) with a primary end-point of PFS and primary comparators (Rd continuous and vs. MPT) (70). Results proved that Rd continuous therapy had improved PFS compared to Rd18 and MTP and a superior OS compared to MPT but not to Rd18. Rd continuous therapy had higher response rates and a manageable safety profile. The trial, therefore, supports the continued use of Rd in transplant-ineligible NDMM patients.

In very old patients, monitoring for AEs from prolonged use of steroids is crucial. All patients receiving Rd should receive anti-thrombosis prophylaxis. Aspirin is sufficient in most patients; however, in high-risk patients, low-molecular-weight heparin or warfarin is required (15). Delforge et al. analyzed the effect of age on Health-Related Quality-of-Life outcomes in patients with NDMM in the FIRST trial. The benefit of continuous Rd therapy on PFS was well-maintained in patients >75 years and demonstrated statistically significant improvement for health utility (71).

Rd use in patients ≥65 years was further analyzed in a large multicenter phase III trial. 662 transplant-ineligible patients were randomly assigned to receive low-dose lenalidomide-dexamethasone (Rd) or lenalidomide-prednisone plus melphalan (MPR) or cyclophosphamide-prednisone-lenalidomide (CPR) for induction. In all patient groups, the addition of an alkylating agent to lenalidomide-steroid combination provided no additive benefit in PFS and OS beyond Rd alone. The cumulative grade ≥3 toxicity was neutropenia, and grade ≥3 non-hematologic toxicities were similar among arms and were mainly infections, constitutional, and cardiac. Rd was associated with lower toxicity. This analysis's safety and efficacy data suggested triplet regimens in patients ≤75 years and doublet regimens for patients >75 years (39).

Recently the VRd regimen was modified for older patients. A Phase II study using a dose-attenuated “RVd-lite” regimen in transplant-ineligible myeloma patients aged 65–91 (median 72) demonstrated that similar benefits of an effective drug combination could be achieved in older transplant-ineligible patients as in younger patients with adequate dose modification without compromising efficacy. RVd-lite improved ORR at 86%, median PFS of 35.1 months; median OS was not reached, with a significantly low discontinuation rate at 4%. Fatigue was the most common grade 1 or 2 toxicity. Grade ≥3 toxicities included hypophosphatemia, neutropenia and rash with minimal grade 3 peripheral neuropathy. Because RVd-lite is well-tolerated and highly effective, it is an attractive alternative for transplant-ineligible patients unable to tolerate the standard VRd regimen (72) without compromising efficacy.

Pomalidomide is a second-generation IMiD agent that showed promising efficacy for RR MM refractory to previous treatment with lenalidomide and bortezomib. A retrospective analysis of 14 RR MM patients aged 58–84 years (median 72 years) investigated the tolerability and safety of pomalidomide. Patient data were compared among three age groups: <70, 70–75, and >75 years. Pomalidomide was well-tolerated, particularly in older patients. However, in patients with poor performance status, tolerability to pomalidomide was low, demonstrating the importance of determining tolerability in the early phases of treatment. The most frequently reported grade 3/4 hematological AEs were neutropenia, anemia and thrombocytopenia. Pomalidomide has the convenience of oral administration and is safe and efficacious even in patients with severe renal impairment. Additionally, it can be administered in late-stage RR MM patients in a real-world clinical setting. In older patients or patients with poor performance status, the dosage must be individualized or given cautiously (73).

The combination of pomalidomide, cyclophosphamide and dexamethasone (PCd) for older patients with RR MM was investigated in the KMMWP-164 study. Fifty-five transplant-ineligible RR MM patients aged 64–86 years (median 73.3) with failed PI and IMiD therapies were included in the study. Results showed improved ORR and PFS; however, many AEs were reported, including infection and pneumonia. Although PCd yields higher response rates and improved survival outcomes, it is associated with frequent and severe toxicities. Dose modification is recommended with initial PCd treatment (74).

### Combinational Therapies With Monoclonal Antibodies

Combinational therapies with IMiDs, PIs, and mAbs, are coming into the limelight for transplant-ineligible patients and refractory/relapsed disease. Daratumumab, the first-in-class fully human mAb, binding CD38, was initially approved after at least 1 line of therapy (75). The safety and tolerability of daratumumab combination therapy were demonstrated in the phase III ALCYONE study of 706 patients aged 40–91, newly diagnosed MM, ineligible for ASCT to receive 9 cycles of daratumumab plus VMP (D-VMP) or VMP only. Results showed prolonged PFS 71.6 vs. 50.2%, improved ORR 90.9 vs. 73.9% and reduction in the risk of disease progression with the mAb combination leading to its approval in transplant-ineligible NDMM patients. However, the daratumumab combination was associated with higher hematologic AEs than the control group; grade 3/4 infections 23.1 vs. 14.7%, neutropenia 39.9 vs. 38.7%, and a low treatment discontinuation rate (76). Generally, combining daratumumab with VMP did not increase overall toxicity.

Subsequent trials such as the phase III MAIA study evaluated the combination of daratumumab plus Rd vs. Rd alone for transplant-ineligible patients. The study randomized 737 patients (368 to D-Rd; 369 to Rd), aged 45–90. Results showed improved response rates with CR 47.6 vs. 24.7%, VGPR 79.3 vs. 53.1% with a 30-month PFS of 71 vs. 56% with Rd alone (*P* < 0.0001) favoring the monoclonal combination. Higher rates of grade 3/4 pneumonia 13.7 vs. 7.9%, neutropenia 50.0 vs. 35.3% and leukopenia 15.1 vs. 10.7% were observed in the D-Rd arm with a low treatment discontinuation rate owing to the favorable safety profile of D-Rd (77). The study supports the addition of daratumumab to standard care regimens in patients with NDMM ineligible for transplant (78). The GRIFFIN study of D-RVd (patients age 18–70) and the CASSIOPEIA study of D-VTd (patients age 18–65) have demonstrated the clinical benefit of the addition of daratumumab to triplet regimens in transplant-eligible NDMM patients, showing the all-inclusive potential of daratumumab in various patient categories (79, 80).

### Risk Stratification and Regimen Modification

Treating older adults incorporates the selection of regimen and schedule depending on patient fitness assessment, comorbidities, disease characteristics, and understanding the goal of treatment. IMWG frailty index categorizes patients as fit (score 0), unfit (score 1) and frail (score ≥2) with patients >80 years regarded as frail in clinical trials. R-MCI categorization is between fit (index ≤ 3), intermediate (index 4–6), frail patients (index > 6). Because no clinical trials tailored for transplant-ineligible patients with high-risk disease have been undertaken, the information used for the treatment of this population is derived only from a subgroup analysis of clinical studies. Despite not having a clear survival benefit algorithm available for high-risk patients, the IMWG recommends a combination of PIs with IMiDs and dexamethasone such as RVd-lite in this setting.

According to the 2020 myeloma updates on risk stratification, high-risk MM is defined by the presence of del (17p), t (4;14), t (14;16), t (14;20), gain 1q, or p53 mutations, with the presence of any two high-risk factors considered as double-hit myeloma, and of any three as triple-hit myeloma. Standard risk disease is associated with the presence of t (11;14), t (6;14) and or trisomies and low-risk disease characterized by a normal cytogenetic profile. Cytogenetic abnormalities are associated with unique clinical and immunological characteristics of MM at diagnosis and may influence the response to novel agents (81).

In high-risk patient's ineligible for transplant, VRd is administered for ~8–12 cycles for induction, followed by dual maintenance consisting of lenalidomide and bortezomib every other week if safely tolerated. For patients with contraindications to bortezomib, ixazomib once per week in place of bortezomib is recommended. DRd is a favorable alternative to VRd. In standard-risk patients, VRd is administered as initial therapy for 8–12 cycles followed by maintenance with lenalidomide. DRd is an alternative to VRd but at a risk of toxicity of long-term triplet regimen. A triplet regimen is required for refractory disease with the choice of regimen varying according to the consequential relapse (82).

In older patients with high-risk cytogenetics, treatment with combinational novel agents is likely to improve response rates (25, 83). Combining a PI with lenalidomide/pomalidomide and dexamethasone reduces the adverse effect of t (4;14) and del(17p) on PFS in NDMM. In recent research, carfilzomib plus cyclophosphamide and dexamethasone (KCd) for both induction and maintenance therapy in transplant-ineligible NDMM mitigated the poor prognosis carried by high-risk cytogenetics and yielded similar PFS and OS in high-risk and standard-risk patients showing that prolonged carfilzomib use is a beneficial alternative for high-risk MM disease (84). Carfilzomib, with lenalidomide plus dexamethasone (KRd), has demonstrated high efficacy and favorable safety profiles eliciting durable responses including, MRD negativity and increased PFS and OS rates in both transplant-eligible and -ineligible patients, across age subgroups and regardless of cytogenetic risk (85).

Thalidomide does not abrogate high-risk cytogenetic abnormalities. Currently, no conclusive clinical data is available for older or frail patients. Bortezomib partly overcomes the adverse effect of t (4, 14) and possibly del(17p) on CR, PFS, and OS. In transplant-ineligible patients with high-risk cytogenetics, no data is available to suggest that lenalidomide as a single agent may improve outcomes. However, VMP may partly restore PFS in this subgroup of patients. Pomalidomide with dexamethasone has shown promising results in RR MM with the high-risk abnormality del(17p) (24). A subgroup analysis of the CASTOR study evaluated D-Vd vs. Vd for RR MM based on cytogenetic risk. D-Vd achieved deep response rates and higher MRD negativity rates than Vd, regardless of cytogenetic risk (86).

The treatment of older patients with MM can be complicated by underlying comorbidities such as diabetes, coronary heart disease, and HTN. CKD, also associated with HTN, progresses to end-stage kidney disease within 3 months after diagnosis in 65% of patients with cast nephropathy. Treatment-related mortality and morbidity are higher in older patients with CKD. Bortezomib-based regimens achieve improved CR and complete renal rates in older patients with renal impairment and are recommended in this setting (87). Lenalidomide should be used with caution in CKD with mandatory dose reduction because the drug is renally cleared. Thalidomide can be used at doses of 50–100 mg as tolerated. Renal transplantation alone without treating MM is not recommended because of recurrence. A recommended treatment-free remission of 3–5 years is advised before renal transplantation. In patients with reduced GFR, denosumab is preferred over bisphosphonates to reduce the risk of skeletal events. Dialysis is indicated in patients with critically low GFR (estimated GFR 15 ml per min per 1.73 m2 or below) or with symptomatic uremia. If HTN is the cause or complication of CKD, it should be treated appropriately with lifestyle modification. Supportive treatment, electrolyte correction and withdrawal of nephrotoxic drugs may improve renal function.

Hepatitis B and C Viruses (HBV, HCV) are well-known complications in hematological malignancies during and after cytotoxic chemotherapy. In Asia, where HBV incidence is high, HBV reactivation has been reported with PI and IMiD use. HBV reactivation can cause severe hepatitis and lead to fatal fulminant hepatitis. Antiviral prophylaxis from initiation of MM therapy is recommended and monitoring HBV markers is crucial for older MM HBV-positive patients (88).

### Recommended Regimens and Dose-Modifications

Until recently, the standard care of treatment for older transplant-ineligible MM patients was alkylating agent melphalan combined with prednisone. The development of novel agents widened treatment options for older patients. The goal of treating older patients is decreased toxicity and prolonged survival. To individualize treatment for older patients, consideration of factors that may influence treatment outcome is essential. Disease characteristics, age, frailty, disability and pre-existing comorbidities should be assessed to determine a patient's ability to tolerate treatment (3). Geriatric medicine is important in managing very old MM patients. Patient comorbidities, cultural beliefs and limited access to health care present barriers to appropriate health care. In developed countries, older and very old patients are managed in community facilities, with only a small percentage of them included in trials making it difficult to converge complete data on the optimal patient treatment.

**Figure 1:**
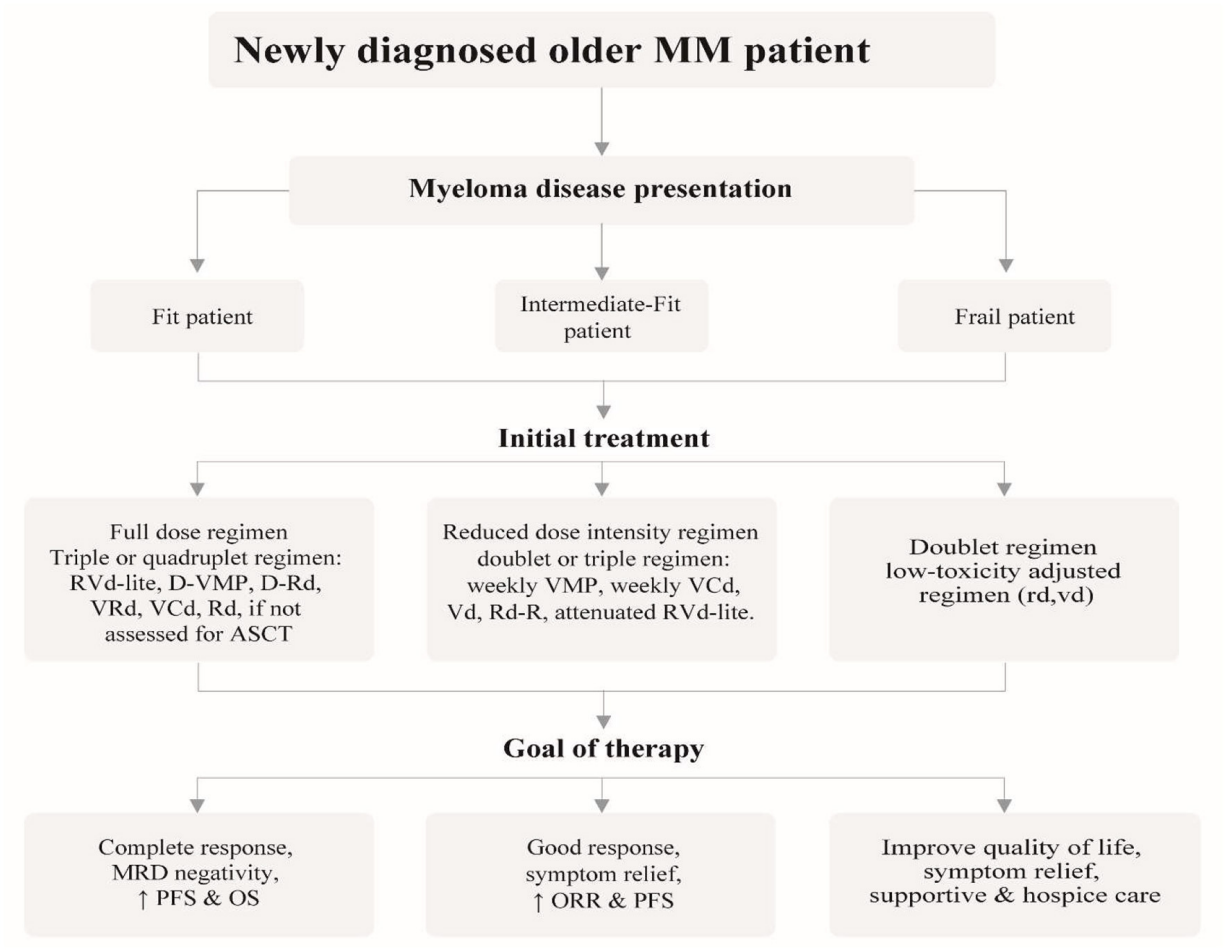
Recommended approach for the management of newly diagnosed older myeloma patients, outlining the different treatment strategies based on patient fitness status and desired goal of therapy.

Clarifying the goal of treatment to older and very old patients is crucial as they are likely to have expectations related to the quality of life, physical independence and goals related to their friends and family. Patients should be allowed to decide on the choice of treatment considering family burden such as financial costs and time spent in hospital against a realistic assessment of the potential benefits that might be derived from that treatment. The discussion of treatment should include all alternatives, including palliative and hospice care (5). Ensuing adapted dose regimens and monitoring treatment-related toxicity guarantee ideal efficacy-safety balance for older patients. Figure 1 gives an intuitive approach to treating older newly-diagnosed myeloma patients based on fitness and risk stratification including the goal of therapy.

#### Induction Therapy

The goal of induction therapy in MM is rapid disease cytoreduction. In fit NDMM older patients, a triplet or quadruplet regimen that includes both PI and IMiD is recommended. RVd-lite is preferred and is currently the standard of care. Monoclonal antibody combinations are useful alternatives reported to improve PFS and ORR such as the newly approved DRd and the quadruplet regimen D-VMP. The treatment of older MM patients is rapidly evolving. Still, lenalidomide's significant activity and manageable safety profile deem it important in older patients unfit for ASCT (89). Therefore, Rd remains a front-line regimen. VRd (8–12 cycles), VCd are suitable alternatives in patients not assessed for ASCT. In patients unable to tolerate triplet regimens such as intermediate-fit patients, Rd and Vd improved response rates and ORR, respectively. A useful strategy is to utilize standard therapy with dose or schedule modifications like weekly VMP, Weekly VCd, RVd-lite, and Rd-R.

In frail patients, dose-adjusted regimens with rd and vd are recommended or consider only supportive care or hospice for frail patients who present aggressively (9, 89, 90). Rd gives an optimal option in frail patients without a caregiver and living far from the hospital because of its advantage of oral administration and long-term tolerability. In frail older patients unable to tolerate RVd at an attenuated dose, DRd, Rd, or Vd can be considered in that order of preference (91).

#### Consolidation Therapy

Consolidation therapy aims to deepen the response to the initial induction therapy. In patients ineligible for ASCT, a single- or multi-drug regimen can be administered, with preference given to lenalidomide (89).

#### Maintenance Therapy

Maintenance therapy after remission (≥CR) prolongs PFS and OS, even in older patients with high-risk cytogenetic profiles. Maintenance with lenalidomide is preferred after induction as it provides survival benefits in both fit and unfit patients for ASCT. Lenalidomide maintenance is effective, well-tolerated and convenient (92). However, lenalidomide maintenance is associated with an increased risk of hematologic AEs and hematologic and solid secondary primary malignancies (91).

Bortezomib is another option for maintenance therapy. In the upfront trial, 25 weeks of bortezomib maintenance after induction in patients ineligible for transplant improved response rates, including CR and ≥ VGPR (10). Bortezomib administered every other week has shown to improve OS. Dual maintenance with lenalidomide and bortezomib may benefit a selected group of patients with high-risk cytogenetics (89). In patients unable to tolerate long-term bortezomib, ixazomib maintenance is an appropriate alternative (93). Although the benefit of maintenance therapy is established, data on optimal duration is unavailable.

#### Relapsed Refractory Disease

The goal of treatment in RR MM is to induce as deep a remission as possible to achieve improved PFS. Treating RR MM patients remains a challenge because RR MM continuously evolves and develops resistance to treatment. Patients may face a difficult decision-making process at every phase of their disease. Clinicians may face a difficult-to-treat RR MM in frail, older or very old patients with comorbidities and failed subsequent lines of therapy. Sequencing of therapy at each subsequent relapse is individualized based on patient status, comorbidities, previous treatment AEs, prior therapies, time and aggressiveness of relapse, duration of previous responses, and the availability of any new cytogenetic abnormalities. Generally, a triplet regimen is preferred over a doublet. If patients relapse while receiving lenalidomide or bortezomib maintenance, a triplet regimen with one new class of agents is advised. An alternative would be a second-generation agent from the same class of drugs initially given.

Treatment algorithms following relapse after one to three lines of therapy have been created using results of previous trials conducted in relapsed refractory disease. In fit patients with disease relapse during lenalidomide maintenance, carfilzomib-pomalidomide-dexamethasone (KPd) (94) and pomalidomide-bortezomib-dexamethasone (PVd) (95) are preferred options. KRd is favored in fit patients with disease relapse during bortezomib maintenance. The phase III ASPIRE trial investigated the benefit/risk profile of KRd vs. Rd in relapsed myeloma patients with high-risk cytogenetics with ≥1 prior line of therapy and prior bortezomib exposure. Results yielded an improved PFS hazard ratio with continuous CR at 18 months following 18 cycles of KRd than Rd alone showing the potential clinical benefit of continuous carfilzomib (96). In fit patients not receiving maintenance therapy during relapse, KRd and DRd are good options, whereas ixazomib-lenalidomide-dexamethasone (IRd) and elotuzumab-lenalidomide-dexamethasone (ERd) are preferred in frail patients.

Pomalidomide-dexamethasone (Pd) is a useful option for RR MM patients with failed lenalidomide and bortezomib therapy with two prior therapies (91). Promising results were reported in the IFM 2010-02 trial with Pd use in relapsed refractory myeloma in patients with high-risk del(17p) and t (4;14) (97). The ELOQUENT-3 trial demonstrated the enhancement of Pd efficacy in combination with elotuzumab (98). Daratumumab-Pd, a PI with panobinostat, carfilzomib-Cd and pomalidomide-Cd are alternative options for relapsed refractory myeloma. In frail patients with relapse during bortezomib maintenance, DRd, IRd, or ERd are effective. Daratumumab-based regimens can be attempted at the second or third line (91). Following relapse after four or more lines, patients can be considered for clinical trials or retreatment with previous agents while providing the best supportive care.

The recent phase III CANDOR trial randomly assigned 466 patients with RR MM to receive carfilzomib, dexamethasone and daratumumab (KdD) or carfilzomib and dexamethasone (Kd). In patients ≥75 years, dexamethasone was reduced to 20 mg weekly. Grade 3 AEs were higher in the KdD group, including thrombocytopenia, anemia, hypertension, pneumonia, fatigue, neutropenia and lymphopenia. The AEs were consistent with the known safety profiles of each agent suggesting that combining daratumumab to carfilzomib does not result in additional toxicity. KdD achieved prolonged PFS and deeper responses compared to Kd and had a favorable benefit/risk profile (99).

Although MM remains an incurable disease, multi-drug combinations can prolong survival in both untreated and relapsed MM in older adults. Individual patient characteristics and comorbidities play a significant role when determining the treatment regimen for older adults, thus emphasizing the need for an individualized approach (90). Managing older MM patients in resource-limited facilities such as developing countries with challenges of diagnosis and treatment is a dilemma to clinicians. Late diagnosis due to lack of modern diagnostic equipment and novel therapies results in most facilities preferring a palliative approach. In developing countries, MP remains the definitive treatment against the standard “RVd lite” preferred in developed countries. The high cost and unavailability of ASCT in addition to the challenges mentioned result in complications, poor prognosis and survival of MM patients in these regions (100).

### Toxicity Management and Supportive Care

Older patients have a higher incidence of AEs and drug discontinuation. Therefore, appropriate and adequate supportive care and early identification of complications and toxicity are vital. Multidisciplinary teams and specialists should be involved in patient care such as palliative care, orthopedics, and interventional radiology. IMiDs and advancing age increase the risk of VTE. Full-dose anticoagulation with low-molecular-weight heparin prophylaxis or warfarin should be considered in high-risk patients with low-dose aspirin, 81 to 325 mg daily as a suitable alternative in patients with one risk or no risk factors. In very old patients with a high risk of VTE, bleeding risk should be considered before initiating anticoagulant prophylaxis. This is because cancer patients have a higher risk of bleeding than non-cancer patients when on anticoagulant prophylaxis. Aspirin carries a lower risk of bleeding complications than do anticoagulants.

Older patients are prone to infections due to immune compromise. The dysfunction of plasma cells, corticosteroid use and treatment-related neutropenia increase the risk of infection and death secondary to infection in the first 3 months after diagnosis. Therefore, antimicrobial prophylaxis should be initiated during induction and administered based on the total steroid dose prescribed. A one-time pneumococcal vaccine should be administered at diagnosis, in addition to yearly influenza vaccination. Growth colony-stimulating factor and a myeloid growth factor can be used to circumvent severe neutropenia. Prophylactic antiviral therapy for herpes zoster is indicated in patients on PIs and daratumumab. The therapy should be administered for 3 months from the commencement of PIs and daratumumab. Patients on bortezomib can receive acyclovir 400 mg twice daily or valacyclovir 500 mg daily (101).

Anemia related to MM or worsened by chemotherapy can be managed with red cell transfusion, erythropoietin-stimulating agents and iron infusion. Patients experiencing pain from symptomatic lytic lesions may benefit from a short course of radiotherapy, analgesics, and rarely orthopedic surgery. Supplementation with Calcium and Vitamin D is advised to maintain calcium homeostasis. Bisphosphonates reduce the risk of skeletal-related events, decrease morbidity, and prolong PFS when used synergistically with anti-myeloma agents. They are strongly recommended at the initiation of anti-myeloma therapy. The bisphosphonates, zoledronic acid or pamidronate administered intravenously every 4 weeks with initial therapy should be continued in all patients with active disease (102). In patients with a VGPR and CR, bisphosphonates can be discontinued after 2 years (9).

With bisphosphonate therapy, preventive strategies and dosage reduction should be considered to avoid renal toxicity and osteonecrosis of the jaw. Denosumab, a receptor activator of nuclear factor kappa B ligand (RANKL) inhibitor, was approved to treat and prevent osteolytic lesions in myeloma. A recent double-blind, randomized controlled trial demonstrated denosumab was non-inferior to zoledronic acid in reducing the incidence of skeletal-related events with an otherwise reduced incidence of renal impairment to half of that seen in patients treated with zoledronic acid (103).

In patients with suspected MM with spinal cord compression, dexamethasone should be initiated before a definitive treatment plan is made. The immediate and prompt commencement of dexamethasone may reverse renal dysfunction in ~50% of patients (104). Corticosteroids may require the use of GI prophylaxis and sometimes even hypoglycemic drugs in patients with diabetes. Surgical care for MM bone disease is mostly adjunctive. Patients with impending fractures should have an early orthopedic evaluation or radiation oncology for the prevention of fractures.

Most older patients suffer chronic pain due to myeloma bone disease, chemotherapy-induced neuropathy and post-herpetic neuralgia. Bisphosphonates, denosumab, and analgesics like opioids, acetaminophen and corticosteroids are administered for pain induced by myeloma-related bone disease. Others like radiotherapy and vertebroplasty are useful pain control techniques. Neuropathic pain can be managed with tricyclic antidepressants, serotonin/norepinephrine reuptake inhibitors like duloxetine or gabapentinoids. Topical analgesics such as lidocaine 5%, capsaicin 8% and antidepressants/anticonvulsants are preferred for post-herpetic neuralgia (105).

## Emerging Future Regimens and Treatment Options

New upcoming agents have widened treatment options for MM. Ongoing trials are currently comparing and incorporating different anti-myeloma agents to establish suitable upfront treatment strategies for older patients.

### Second-Generation Proteasome Inhibitors in MM

Carfilzomib is a second-line, irreversible PI that emerged as a therapeutic option for transplant-ineligible older patients (106). Carfilzomib is cost-effective and likely to take the place of bortezomib in the future due to reduced toxicity profiles with a lower incidence of polyneuropathy (107). The FDA has since approved carfilzomib in the United States to treat patients with RR MM with at least two prior treatments, including bortezomib and an immunomodulatory agent (108). However, carfilzomib should be avoided in patients with pre-existing cardiac disease.

In the phase I/II escalation study to investigate the safety and efficacy of carfilzomib plus MP (KMP) in patients aged ≥65 years ineligible for ASCT, KMP showed favorable efficacy and toxicity profiles in NDMM older patients (109). The phase I IFM-201203 trial demonstrated improved response rates, safety and efficacy profiles with weekly carfilzomib plus MP for patients older than 75 with only three dose-limiting toxicities reported (110).

A multicenter, phase II trial evaluated the safety and efficacy of carfilzomib combined with cyclophosphamide and dexamethasone (KCd) in NDMM patients ≥65 years, ineligible for ASCT. 58 patients received nine 28-day cycles followed by carfilzomib maintenance until progression or tolerance. Results showed that 95% patients achieved a PR, 71% patients a VGPR, 49% patients a near-complete response (CR/nCR) and 20% patients a stringent complete response (sCR). Reported AEs were a few grade 3/4 infections, 1 grade 3 VTE and no cardiac toxicities. The study proved that KCd achieved excellent CR with high safety and efficacy profiles and was well-tolerated with lower drug discontinuation rates in older patients ineligible for transplant (8, 38).

Recently, a secondary study of phase III ASPIRE study showed that carfilzomib combined with Rd was more effective than Rd alone for relapsed MM. This analysis showed improved median PFS in the carfilzomib group, in patients <70 years, 28.6 months' vs. 17. 6 months and ≥70 years, 23·8 months' vs. 16·0 months. ORR was improved in the carfilzomib group compared to the control group; ORR for patients <70 years was 86·0 vs. 66·9%; for patients ≥70 years, 90·3 vs. 66·1%. KRd was associated with grade ≥3 cardiovascular AEs commonly among patients ≥70 years compared with patients <70 years old. KRd has a favorable benefit/risk profile in RMM, including older patients ≥70 years (111).

Ixazomib, a second-generation, oral reversible PI, has shown promising anti-myeloma effects in previously untreated RR MM. In over 40 countries, including the US, EU, Canada and Japan, Ixazomib (Ninlaro) with Rd for treating MM in patients who undergo one-prior treatment has been approved (112). The approval was supported by findings of the global, phase III, randomized, double-blind, placebo-controlled Tourmaline MM-1 study of 722 patients. The study compared the safety and efficacy of ixazomib plus Rd vs. placebo-Rd in adult patients with RR MM. IRd extended median PFS by at least 6 months compared to the placebo regimen in RR MM (IRd arm 20.6 months vs. placebo-Rd 14.7 months, HR 0.742, P = 0.012) with limited toxicity (113). The ORR was 78.3% in the ixazomib group, with a median duration of response of 20.5 months, 71.5% and 15 months in the control group. Grade 3/4 gastrointestinal toxicities, rash, thrombocytopenia and pneumonia were common in the IRd group. The clinical trial proved the safety and clinical benefit of ninlaro in patients with RR MM. Ninlaro has been deemed effective and safe for use in real-world practice (114).

Ixazomib (MLN9708), in combination with Rd, was investigated in a phase 1/2 study of 65 patients aged 18 years or older (115). Cumulative results showed that 96% of patients achieved ≥PR, 44% with ≥VGPR, and 26% with a CR. In patients with previously untreated MM, IRd was generally well tolerated with only one grade 3 PN. More investigational trials on ixazomib are ongoing; two trials at phase two with 33 patients aged ≥18 years are investigating ixazomib as a monotherapy in relapsed MM that is not refractory to bortezomib (116). Marizomib and oprozomib are new oral and irreversible PIs still in the first phases of clinical development for RR MM treatment. Phase I study with marizomib demonstrated relatively low toxicities with no evidence of neuropathy or thrombocytopenia. Similarly, two-phase Ib/II studies with oprozomib demonstrated a tolerable safety profile with a low incidence of neuropathy (117).

### Novel Monoclonal Antibodies in MM

Elotuzumab and daratumumab (mAbs) are promising agents that have shown improved outcomes in patients with RR MM. Elotuzumab, an anti-CS1 (CD2 subunit 1) mAb, targets SLAMF7 signaling (lymphocytic activation molecule family member 7) highly expressed in normal plasma, MM cells and natural killer cells (118). The safety and tolerability, pharmacokinetics and pharmacodynamic properties of elotuzumab were evaluated in a multicenter phase I trial as the first-in-human study of elotuzumab. A standard 3+3 design was used to determine the maximum-tolerated dose. 35 patients aged ≥18 years with RR MM were treated with intravenous elotuzumab (dose range 0.5–20 mg/kg every 2 weeks). AEs reported were infusion-related such as cough, headache, back pain, fever and chills. Generally, elotuzumab was well-tolerated without dose-limiting toxicity, justifying a further investigation of combinational therapy rather than as monotherapy (119).

Subsequent studies have investigated combinational therapies of elotuzumab. In the phase IB part of the 1703 study, elotuzumab at escalating doses of 5, 10, and 20 mg/kg with Rd was investigated for treating RR MM. Twenty-nine patients were included in the study with a reported median of 3 prior therapies. Results showed ORR 82% for 28 patients, CR 4% for 1 patient, VGPR 43% for 12 patients and PR 36% for 10 patients. The 20 mg/kg cohort was expanded because no obvious dose-limiting toxicities were observed during dose escalation (120). The second series of the study, phase II of the 1703 study evaluated elotuzumab 10 or 20 mg/kg through randomization with Rd for RR MM. Seventy-three patients with a history of 1–3 prior lines of therapy were included. In the group that received 10 mg/kg, ORR was 92% and 76% for the group receiving a tentatively higher dose of 20 mg/kg. On average ORR was 84%, with 3 (4%) stringent CR, 7 (10%) CR, 31 (42%) VGPR and 20 (27%) PR. Both studies proved that the combination of elotuzumab with Rd was well-tolerated with satisfactory safety and efficacy profiles (121).

The randomized phase III ELOQUENT-2 study evaluated the safety and efficacy of elotuzumab plus Rd (ERd) compared to Rd for RR MM. ERd reduced the risk of progression/death by 30% compared to Rd with a favorable PFS of 26% (ERd) vs. 18% (Rd), improved ORR 79% (ERd) vs. 66% (Rd) after an extended 3-year follow-up. Interim OS was 91 vs. 83% at 1 year, 73 vs. 69% at 2 years and 60 vs. 53% at 3 years favoring ERd. OS benefit was consistent across all subgroups, including patients ≥75 years with prior bortezomib exposure and refractory to recent therapy. AEs were comparable in both arms with anemia and neutropenia being the most common grade 3/4 AEs. The addition of elotuzumab to Rd provided a durable and significant improvement in efficacy with minimal incremental toxicity despite longer follow-up (122). Similar safety profiles are reported in the recent phase II study in Japan, which demonstrated that ERd was an effective, well-tolerated front-line treatment in NDMM patients ineligible for transplant (123).

Daratumumab is a CD38-targeting, human IgG1κ mAb approved for the treatment of RR MM in patients with progressive disease despite the use of PIs and IMiDs. Myeloma cells overexpress CD38 (124); therefore, daratumumab may be a key agent in refractory disease (125). In patients with heavily pretreated and refractory MM, daratumumab, as monotherapy, showed favorable safety and efficacy profiles, as demonstrated in a phase I-II trial (126). The efficiency of daratumumab in combination with Rd in RR MM was investigated in the phase III POLLUX trial. 569 RR MM patients with a median of one prior line of therapy were included in this study. Results showed a higher ORR in the DRd arm compared to the control group (92.9 vs. 76.4%, HR 0.37, *P* < 0.001) and a prolonged PFS (63% reduced risk in disease progression). The most common grade 3/4 AEs during treatment were neutropenia (51.9 vs. 37.0%), thrombocytopenia (12.7 vs. 13.5%) and anemia (12.4 vs. 19.6%). Daratumumab infusion-related reactions occurred in 47.7% of patients and were mostly grade 1 or 2. (127). DRd had manageable AEs consistent with the known toxicities of each agent with low treatment discontinuation rates.

Findings of the POLLUX trial were comparable to the phase III CASTOR trial of DVd. 498 RR MM patients with a median of two prior therapy lines were included. Higher ORR was seen in the DVd group compared to the control group (82.9 vs. 63.2%, HR 0.39, *P* < 0.001) with extended PFS (61% reduced risk in disease progression) (128). Recent reports by Laubach, J. proved that daratumumab with Rd was effective for previously untreated and treated MM in transplant-ineligible patients (129).

## Conclusion

MM is becoming an increasingly prevalent disease in older adults with poorer outcomes despite novel therapeutic regimens. Aging is inevitable and is associated with increased comorbidities and poor performance indicators contributing to the underrepresentation and exclusion from clinical trials. The diagnosis of MM in the very old is usually delayed, resulting in deferred treatment and progressive symptoms. Clinical signs and symptoms presented by the very old can be attributed to other etiologies leading to uncertainty of a precise diagnosis of myeloma. Thorough and timely investigation measures are necessary to exclude diseases that mimic myeloma in older adults. While performing geriatric assessment in everyday practice is time-consuming and challenging, it should nonetheless, be mandatory.

**Figure 2:**
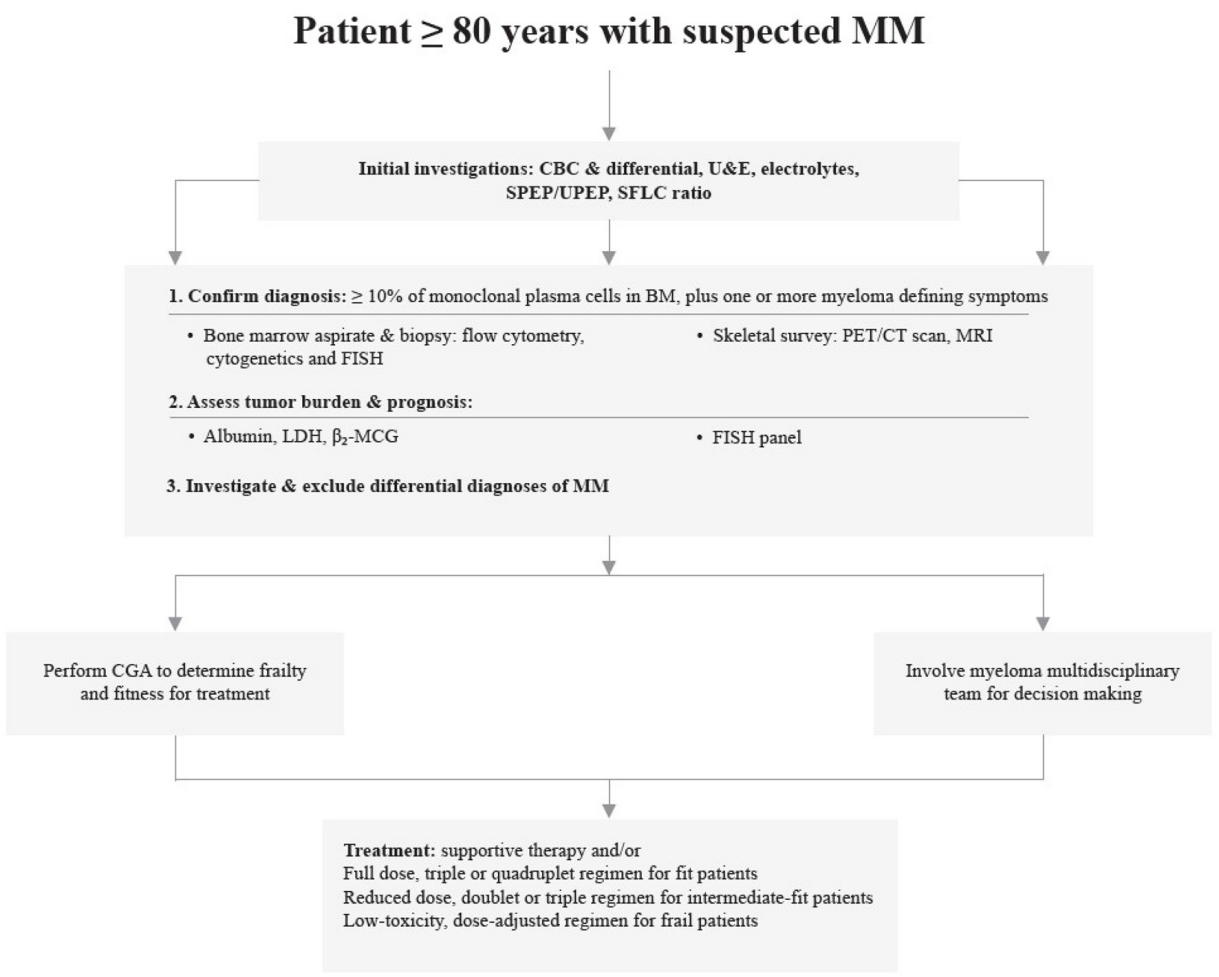
A simplified representation pragmatic to the diagnosis and management of multiple myeloma in patients ≥80 years.

Treating MM in subjects >80 years requires a tailored and adjusted approach. The selection and aggressiveness of treatment should be made using a multidisciplinary team approach. Clinical trials have demonstrated the benefit of novel anti-myeloma agents in older patients. Fit older patients are likely to benefit from a triplet or quadruplet regimen; RVd-lite, Rd, or VCd and VRd are the recommended standard of care for this group. The monoclonal antibody combinations, DRd and D-VMP are also useful options. In frail patients, dose-adjusted rd and vd are recommended or supportive care with hospice for patients with aggressive disease. Upcoming novel agents such as second-line PIs and monoclonal antibodies have demonstrated low-toxicity, good tolerability and safety profiles, making them beneficial prospective agents for treating older patients. Figure 2 demonstrates a simplified representation of a pragmatic approach to diagnosing and managing multiple myeloma in older patients.

## Take Home Message

The incidence of multiple myeloma in older patients is anticipated to rise due to an aging population. As such, clinicians need to acquire knowledge for effectual management of challenges likely to be faced in treating myeloma in older adults.

## Author Contributions

NK performed literature research and review and wrote the final draft of the manuscript. GC revised the final manuscript. FZ conceived and revised the final manuscript. All authors read and approved the final manuscript.

## Conflict of Interest

The authors declare that the research was conducted in the absence of any commercial or financial relationships that could be construed as a potential conflict of interest.

## Abbreviations

ADL: Activities of Daily Living
AEs: Adverse Events
ASCT: Autologous Stem Cell Transplant
CGA: Comprehensive Geriatric Assessment
CCI: Charlson Comorbidity Index
CR: Complete Response
ECOG: Eastern Cooperative Oncology Group
EFS: Event-Free Survival
FCI: Freiburg Comorbidity index
IADL: Instrumental Activities of Daily Living
IMiDs: Immunomodulatory Drugs
KPS: Karnofsky Performance Status
mAbs: Monoclonal Antibodies
MGUS: Monoclonal Gammopathy of Undetermined Significance
MM: Multiple Myeloma
OS: Overall Survival
ORR: Overall Response Rate
PIs: Proteasome Inhibitors
PN: Peripheral Neuropathy
R-IMWG: Revised-International Myeloma Working Group
R-ISS: Revised International Staging System
RR MM: Relapsed and/or Refractory Multiple Myeloma
R-MCI: Revised-Myeloma Comorbidity Index
SMM: Smoldering Multiple Myeloma
VGPR: Very Good Partial Response
VTE: Venous Thromboembolism.
